# Superiority of Multiple-Joint Space Width over Minimum-Joint Space Width Approach in the Machine Learning for Radiographic Severity and Knee Osteoarthritis Progression

**DOI:** 10.3390/biology10111107

**Published:** 2021-10-27

**Authors:** James Chung-Wai Cheung, Andy Yiu-Chau Tam, Lok-Chun Chan, Ping-Keung Chan, Chunyi Wen

**Affiliations:** 1 Department of Biomedical Engineering, Faculty of Engineering, The Hong Kong Polytechnic University, Hong Kong, China; andy-yiu-chau.tam@connect.polyu.hk (A.Y.-C.T.); lc-justin.chan@connect.polyu.hk (L.-C.C.); 2 Research Institute for Smart Ageing, The Hong Kong Polytechnic University, Hong Kong, China; 3 Department of Orthopaedics and Traumatology, Queen Mary Hospital, Hong Kong, China; lewis@ortho.hku.hk

**Keywords:** knee osteoarthritis, deep learning, automatic measurement, joint space width, musculoskeletal disorders, Kellgren-Lawrence grade

## Abstract

**Simple Summary:**

Minimum-joint space width (JSW) is a prevalent clinical parameter in quantifying the joint space narrowing condition in knee osteoarthritis (KOA). In this study, we propose a novel multiple-JSW measurement, which is estimated by a deep learning-based model in an automated manner. The performance of the proposed automated measurement is found to be superior to the conventionally used minimum-JSW in the severity classification and progression prediction of KOA owing to the additional information of the joint space morphology encoded in the new approach. It is further demonstrated that the deep learning-based approach yields comparable performance as the measurement by radiologists. The approach presented in this work may lead to the development of a computer-aided tool for clinical practitioners that could facilitate the KOA diagnosis and prognosis with the fully automated, accurate, and efficient computation of the joint-space parameters.

**Abstract:**

We compared the prediction efficiency of the multiple-joint space width (JSW) and the minimum-JSW on knee osteoarthritis (KOA) severity and progression by using a deep learning approach. A convolutional neural network (CNN) with ResU-Net architecture was developed for knee X-ray imaging segmentation and has attained a segmentation efficiency of 98.9% intersection over union (IoU) on the distal femur and proximal tibia. Later, by leveraging the image segmentation, the minimum and multiple-JSWs in the tibiofemoral joint were estimated and then validated by radiologist measurements in the Osteoarthritis Initiative (OAI) dataset using Pearson correlation and Bland–Altman plots. The agreement between the CNN-based estimation and radiologist’s measurement of minimum-JSWs reached 0.7801 (*p* < 0.0001). The estimated JSWs were deployed to predict the radiographic severity and progression of KOA defined by Kellgren-Lawrence (KL) grades using the XGBoost model. The 64-point multiple-JSWs achieved the best performance in predicting KOA progression within 48 months, with the area-under-receiver operating characteristic curve (AUC) of 0.621, outperforming the commonly used minimum-JSW with 0.554 AUC. We provided a fully automated radiographic assessment tool for KOA with comparable performance to the radiologists and showed that the fine-grained measurement of multiple-JSWs yields superior prediction performance for KOA over the minimum-JSW.

## 1. Introduction

Knee Osteoarthritis (KOA) is a prevalent musculoskeletal disease that is a leading cause of chronic pain and disability in older adults. Clinical diagnosis of KOA relies on plain radiography; the Kellgren-Lawrence (KL) grading system is widely deployed in current practice to subjectively describe the severity and progression of radiographic OA [1]. Joint space width (JSW) is a primary indicator of the integrity of articular cartilage and the severity of KOA [2]. The Osteoarthritis Research Society International (OARSI) atlas [3] has been recently established for feature-specific measurement of JSW; however, similar to the KL-Grade, the subjectivity of individuals becomes detrimental to the repeatability and reproducibility of measurements [4]. There has been a growing interest in the development of automated computer-aided methods for consistent quantification of joint space information on plain radiographs for diagnostics and prognostics of KOA.

One of the most commonly used quantities for the characterization of the radiographic severity of KOA is minimum-JSW. The key to automatic estimation lies in the accurate segmentation of the femur and tibia plateau [1]. The earlier computer-aided approaches were built on traditional methods such as edge detection filters and other statistical algorithms [1,5,6]. Such naive approaches either fail to address the 3D joint structure projection onto 2D images (resulting in the identification of irrelevant bone edges [7] and inaccurate joint space width estimations) or require prior parameterization to roughly localize the bone regions on every image, leading to a lack of automation [8].

Recently, deep learning has emerged with superior performance in extracting sophisticated features from a wide variety of data types [9]. By leveraging such an approach, a number of recent OA studies have yielded great success in the analysis of KOA progression prediction [10], total knee replacement (TKR) prediction based on MRI [11], and human tissue segmentation [12]. However, to our best knowledge, little research has been done in an attempt to identify a smooth, continuous contour of the knee joint for accurate and fine-grained characterization of the tibiofemoral joint space. Wang et al. and Tiulpin et al. leveraged low-cost labels to identify the coarse landmarks instead of a detailed contour of the knee joint [7,13,14]. Meanwhile, Lindner et al. and Thomson et al. employed convolutional neural networks (CNN) to create a bounding box to localize the joint space for subsequent detailed grading [15,16]. The above approaches leverage deep learning or other advanced machine learning methods to generate rough landmarks or regions-of-interest (ROI) for various subsequent applications. However, these coarse-grained localizations do not favor the detailed quantification of joint space features. As a result, a new approach is of great need that is capable of creating fine-grained bone contours with distinguishable relevant edge structures under the 3-D projection in the 2-D radiographic image.

To this end, in this paper, we first develop a deep neural network based on the ResU-Net [15] architecture, which performs automatic segmentation of the tibia and femur. Subsequently, the performance of our ResU-Net approach is compared with the other deep learning-based image segmentation techniques, including CUMedVision [17,18], DeepLabv3 [19,20], and U-Net [21]. Second, with the identification of the tibial and femoral bone contour, pixel-wise quantitative measurements are made to calculate the knee JSW. In particular, apart from the minimum-JSW defined in the medial compartment, the smooth and continuous contours are obtained for the calculation of multiple-JSWs at fixed locations in the tibiofemoral joint. It is inferred that not only the richer one-dimensional information regarding the bone margin could be retrieved, but that together, they could characterize the whole joint shape, which may effectively enhance the detection of radiographic OA, as inspired by Bayramoglu et al.’s recent work [22]. To validate the JSW calculation by our proposed algorithm, we compared our results with the measurements by radiologists from the Osteoarthritis Initiative (OAI) database. Finally, in pursuit of demonstrating the added values of the multiple-JSWs generated by our approach, we compared its prediction prowess towards radiographic severity and progression of KOA, defined by Kellgren-Lawrence (KL) grades with the minimum-JSW measured by our method and clinical practitioners, respectively.

## 2. Materials and Methods

### 2.1. Dataset and Preprocessing

All radiographic images being used were retrieved from the Osteoarthritis Initiative (OAI) database (https://data-archive.nimh.nih.gov/oai, accessed on 1 September 2020). Subjects were recruited (n = 4796) from four centres. The inclusion criteria include men and women of all ethnicities, ages 45–79, with or at risk for symptomatic femoral-tibial knee OA. The exclusion criteria were bilateral end-stage knee OA, inflammatory arthritis, and contraindications that could be found with a 3-Tesla MRI.

For image segmentation, we focus on subjects with bilateral X-ray images from the baseline cohort, which consists of a total of 4216 images from distinct subjects. The subject’s ages ranged from 47–79, with a median of age 61. A KL grade of 0 was discovered in 38.6% of the images, 18.1% had KL grade 1, 26.4% had KL grade 2, 13.7% had KL grade 3, and 3.2% had KL grade 4. In the preprocessing pipeline, the 16-bit DICOM images were first normalized using global contrast normalization and a histogram truncation between the 5th and 99th percentiles. These images were downscaled to 1024 × 1024 pixels for both training and inferencing. Out of the 4216 images, 100 bilateral radiographs (200 knees) were chosen randomly. The masks were being annotated by two authors (A.Y.-C.T. and L.-C.C.) using the Computer Vision Annotation Tool (https://github.com/openvinotoolkit/cvat, accessed on 1 October 2020) and were cross-checked to refine the annotations. Among all the annotated data, 90% were being used for training, while 10% were used for validation. It has been reported that bilateral knee OA patients demonstrated larger interlimb kinematic asymmetry that may lead to different severity of OA among their limbs [23]. As a result, the wearing rate of both legs might be different and could be biased towards one of the legs in the population, thus potentially leading to model overfitting. Given this, horizontal flipping of the X-ray images as a means of data augmentation was employed to improve the model generalization and reduce bias.

### 2.2. Bone Segmentation Using Deep Neural Network

In our automated JSW estimation approach, we first employed a deep learning model to perform bone segmentation on plain radiographic images. To this end, four deep convolutional neural network models, including U-Net [21], CUMedVision [16], ResU-Net [24], and DeepLabv3 [19,20] were selected for producing the segmentations of the X-ray images.

U-Net is a class of neural networks designed for image segmentation that extends the fully convolutional net (FCN) [17] by adding skip connections from encoder layers to decoder layers to facilitate backpropagation through different convolutional layers and, hence, reducing the gradient vanishing problem. This type of network has been widely applied to medical image segmentation, such as knee menisci segmentation from MRI [25] and knee cartilage tracking [26].

CUMedvision is a variant of FCN, which uses multi-level feature fusion to integrate both high-level and low-level features, making it excel in identifying objects with huge size differences on the image [16].

**Figure 1:**
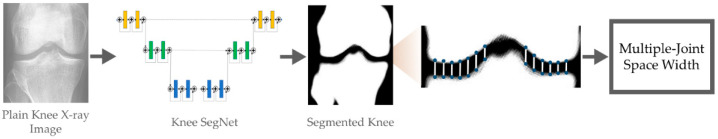
Flow diagram of the multiple-JSW automatic measurement.

On the other hand, ResU-Net is another variant of U-Net, with the addition of residual blocks and skip connections [27]. The residual blocks in ResU-Net further assist in propagating low-level details to higher network layers, thereby facilitating more fine-grained segmentation of objects (Figure 1). Instead of the structure defined in the original work, a low complexity version of ResU-Net using 18 residual layers in place of 50 were applied as the network backbone, which accommodates a lower memory usage for training and better performance in radiographic images.

DeepLabv3 further extends ResU-Net by using dilated convolution, context module, spatial pyramid pooling, etc. [20]. For the hyperparameters and network structures in DeepLabv3 and U-Net, we employed the default settings from PyTorch 1.7.0. While CUMedvision does, in its settings, follow the original paper.

The four selected models are all in an encoder-decoder architecture [17], in which each pixel in the neural network is classified as one of the four categories: femur, fibula, tibia, or background, with a probability between 0 and 1, with a sigmoid function in the output layer. We compared their performance and subsequently selected the best performing model with the highest mean Intersect over Union (IoU) score.

### 2.3. Model Training

In the training procedure of the four models, the deep network is implemented using PyTorch version 1.7.0. The Adam optimizer with a learning rate of 0.001 was used, which provides a tradeoff between training time and accuracy. Weight decay with 1 × 10^−5^ was used, and the early stopping strategy was also applied, which terminates the training when there is no loss improvement for 10 epochs to prevent overfitting. Backpropagation optimizes parameters by minimizing the loss function using a first-order gradient. All four networks use Binary Cross-Entropy (BCE) as the loss function, which aims to maximize the log-likelihood for correct predictions of the classes of each pixel.

(1) $$BCE\left(p\right)=\frac{1}{m}\overset{m}{\underset{i=1}{\sum }}-p^{i}\log\left(p^{i}\right)-\left(1-y^{i}\right)\log\left(1-p^{i}\right)$$

To tackle the issue of limited data, data augmentation was applied to improve the model generalization ability. Histogram normalization was used to maintain consistency across different image sets, which were taken by different observers and equipment. Alongside saturation and contrast jitter, translation and random flipping were also applied in the augmentation process. The rotation and horizontal shifts of the images were ± 5° and ± 10%, respectively.

### 2.4. Quantitative Measurement

**Figure 2:**
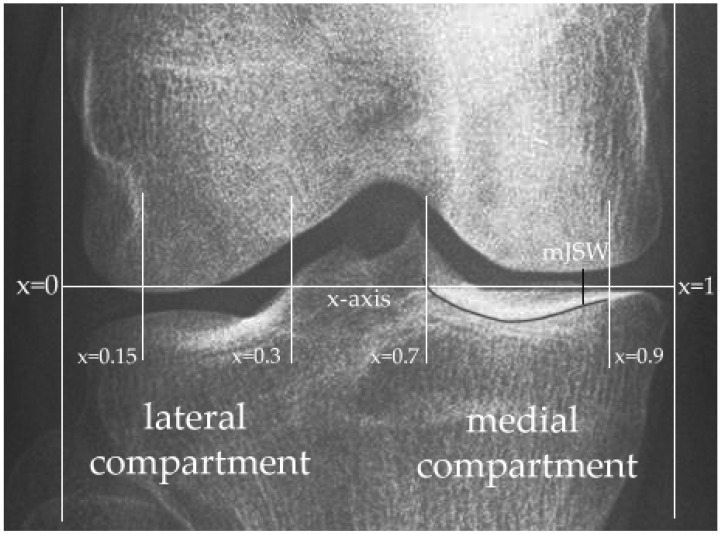
The definition of minimum-joint space width.

Following the output of masks indicating the femur and tibia from the deep neural network, a program for the automated calculation of JSWs was derived. Firstly, contours are being extracted from the femoral and tibial masks generated with Canny filters using the OpenCV 3 package in Python. The horizontal distance of the extracted tibial plateau contour was normalized to a scale of 1. We denote this scale as a variable x (Figure 2). The multi-JSW measurement was calculated in the range x = 0.15~0.30 (lateral compartment) and x = 0.7~0.9 at 0.05, 0.025, 0.0125, and 0.00625 intervals for 8-point, 16-point, 32-point, and 64-point JSWs, respectively. While for the minimum-JSW, pixel distance between all pairs of pixels in the two contour segments of the condyles and tibial plateau were computed in the range x = 0.7~0.9 (medial compartment), and finally, the minimum distance was identified as the minimum-JSW. The measurements were further normalized to a millimeter-scale using the flexion beams. To validate the estimation accuracy, we compared the minimum-JSW calculated by our approach against the radiologists’ measurements from the OAI database. Their correspondence was quantified using Pearson correlation, and the difference was visualized by a Bland-Altman plot [28].

### 2.5. KOA Severity and Progression Prediction

After the development of an automated JSW measuring system, we randomly sampled 1760 bilateral X-ray images from the baseline cohort together with their corresponding KL-grades assessed by the radiologists from the OAI database (those used for training and validation of the segmentation models were excluded) and employed the algorithm to output the minimum-JSW and multiple-JSWs accordingly. We defined the KOA severity using the 5-grade KL-grading system. An XGBoost model, which is a tree-based method capable of capturing nonlinearity within the data structure [29], was trained using the estimated JSWs as input to classify the severity of KOA. The optimal hyperparameters of the model were obtained using grid-search with 5-fold cross-validation. From which, the maximum depth, alpha, and lambda parameters were found to be 30, 1, and 1, respectively.

In the next experiment, the disease progression was defined as an increase in KL-grade from the unaffected (grade 0 and 1) to the confirmed case (grade 2 to 4) within 48 months. Moreover, samples that dropped out of the study before the 48-month follow-up were viewed as data with missing labels and were subsequently ruled out. After the selection, 945 pairs of knees remained. The grid-search procedure with 5-fold cross-validation was performed for this experiment, and the most optimal hyperparameters of the XGBoost model were identified to be maximum depth = 25, alpha = 0.5, and lambda = 1. Both experiments were conducted with an 8:2 train-test split. We evaluated the model performance with the test set using the average macro F1 and average area under the receiver operating curve (AUC) scores with both metrics’ average values obtained by 100 iterations of bootstrapping for severity classification. Meanwhile, the disease progression prediction, average F1, and average AUC scores with 100 iterations of bootstrapping were used. Lastly, in pursuit of comparing the performance of our CNN-based JSW estimation and those measured by radiologists in the prediction of disease severity and progression, we repeated the above experiments using the minimum-JSW and 16-point JSWs from the OAI dataset.

## 3. Results

### 3.1. Reliability of the Annotations

Before training our deep learning model for knee bone segmentation on plain radiographic images, we first assessed the reliability of the annotations in the dataset. The minimum-JSW measurements obtained from the annotated data were further compared to the radiologists’ measurements extracted from the OAI dataset to produce a baseline of interobserver error. The mean interobserver error was 0.483 mm, with a standard deviation of 0.661 mm, and an R^2^ value of 0.9565. The intra-class correlation coefficient (ICC) was used to test the agreement of inter-observer measurement [30]. The ICC between OAI measurement and contour annotator was 0.812, showing that the minimum-JSW measurements have high consistency with the measurements by radiologists.

### 3.2. Bone Segmentation Performance Comparison

**Figure 3:**
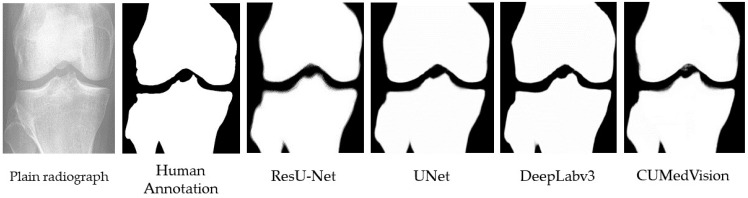
Comparison of masks produced by different semantic segmentation network architectures.

The segmentation accuracies of the four segmentation methods (i.e., CUMed-vision, U-Net, DeepLabv3, and ResU-Net) were compared in Table 1. The segmentation masks produced by the four networks and the annotated mask are shown in Figure 3. Both ResU-Net and DeepLabv3 achieved the highest mean IoU score of 0.989, outperforming the other two candidates. Validation loss of ResU-Net is lower than Deeplabv3 (0.006 < 0.011), showing that the former model outperforms Deeplabv3 in terms of validation loss. Finally, it was noticed that the overfitting score of Deeplabv3 is higher than that of ResU-Net, which indicates its greater tendency of undesirable over-fitting. As a result, the ResU-Net was conceived as the best model in terms of both performance and robustness.

### 3.3. Automated Measurement of Joint Space Width

As the ResU-Net has demonstrated its superiority over the other CNN architectures in this automatic segmentation task on plain radiographs, it was selected as the algorithm to outline the bone contour for subsequent joint space measurements using the OpenCV2 package from python. We then employed the algorithm to segment 4216 X-ray images, then automatically calculated their minimum-JSW in the medial compartment, with estimated numerical values ranging from 0 to 7.16 mm, with a mean of 3.53 mm, and a standard deviation of 1.35 mm. In order to access the validity of our automated estimation, we additionally harvested the JSW measurements by clinical doctors or radiologists of those 4216 images from the OAI dataset. The measurement values ranged from 0 to 7.744 mm, with a mean of 3.68 mm and a standard deviation of 1.36 mm.

**Figure 4:**
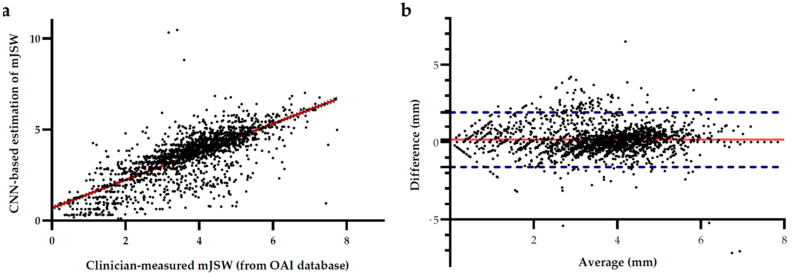
(**a**) Linear regression between minimum-JSW measurements by radiologists or orthopedic doctors (from OAI database) and the proposed deep learning-based automated minimum-JSW estimation approach. The regression line is colored in red with an R^2^ value of 0.6086 and a Pearson correlation of 0.7801 (*p* < 0.0001). (**b**) Bland-Altman plot for comparison between minimum-JSW measurements by radiologists (from OAI database) and our CNN-based automated approach. The Blue dotted line indicates the 95% confidence interval of the difference between the two types of measurement. The red solid line indicates the mean bias of the measurement.

To examine the performance of our proposed deep learning-based automated JSW measurement algorithm, we first performed a linear regression analysis between the minimum-JSW in the medial compartment measured by radiologists, which were obtained from the OAI database and that estimated by our proposed deep learning-based automated method with the automatic measurement method (Figure 4a). A significant correspondence was observed among them with an R^2^ value of 0.6086 and a Pearson correlation of 0.7801 (*p* < 0.0001). Moreover, the Bland-Altman plot [28,31] between the two measurements was also plotted (Figure 4b), which indicated a low mean difference (d = 0.61 mm), while most of the data were within the 95% confidence interval (±1.76 mm) around the mean difference. This indicated a good agreement between the results obtained by the automatic quantitative JSW estimation and measurement by radiologists.

### 3.4. Prediction of KOA Severity and Progression

The accurate JSW measurements enable further study of morphological factors in the severity and progression of OA. KL-grade is a semi-quantitative clinical criterion widely used for the diagnosis of OA, which reflects the severity of OA. The minimum-JSW observes the narrowest points between the tibia and femur plateau in the medial compartment and acts as a monitoring factor for the joint space narrowing (JSN) condition. Nonetheless, this measurement only quantifies the JSW at a single site, which may overlook the whole joint morphological information. Encouraged by our deep learning approach, where continuous contours of the knee joint could be accurately identified, it is possible to measure the JSW at multiple points simultaneously.

In the experiment, 16 points were chosen from both the lateral and medial compartments at a fixed interval. Based on the bone contour identified by our ResU-Net, the algorithm automatically calculated the JSWs at all 16 sites at the same time. Additionally, to demonstrate the added value of using 16-point JSWs over the use of the single-point minimum-JSW, they were compared side-by-side in the prediction of the KL-grade. Table 2 shows that using the 16-point JSWs in place of the minimum-JSW significantly improves both the macro F1 (from 0.311 to 0.402) and AUC scores (from 0.587 to 0.624) in the classification of KL-grades. The measurements by radiologists obtained from the OAI database were also benchmarked with the automatically measured JSWs. Using an unpaired *t*-test, the null hypothesis of having a significant difference of the average AUC between CNN-based automatic measurements and the radiologist measurements was not rejected for the minimum JSW (*p* = 0.1225 > 0.5) while it was rejected for the 16-point JSWs (*p* = 0.0063 < 0.05). Despite having higher prediction scores than the computer-aided estimation in both single-point and 16-points cases, the results still indicated a consistent trend in the classification of KL-grades.

Alongside the 16-point and minimum-JSWs at the baseline were deployed to predict the OA progression defined by the increase in KL-grade from the unaffected to affected condition within the future 48-month period. Significant prediction improvements in both metrics (Table 3) were observed when replacing single-point minimum-JSW with 16-point JSWs, where the macro F1 and AUROC scores increased from 0.484 to 0.544 and 0.554 to 0.583, respectively, while a similar trend was also observed from the radiologist measurements. Using unpaired *t*-tests, the null hypothesis of having significant difference of average AUC between CNN-based automatic measurement and radiologist measurements was not rejected for both the minimum JSW (*p* = 0.898 > 0.5) and 16-point JSWs (*p* = 0.1816 > 0.05).

**Figure 5:**
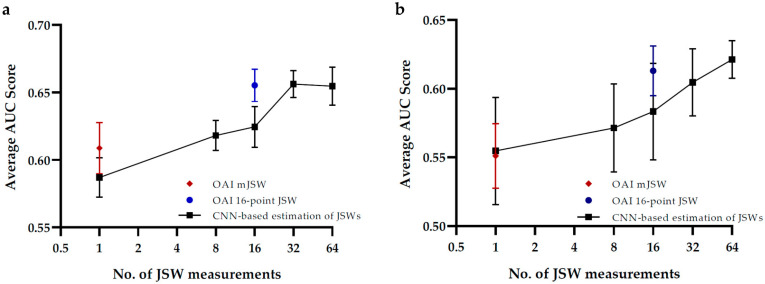
Performance of (**a**) KL-grades classification and (**b**) KOA progression prediction under different numbers of JSWs estimated by our CNN-based approach. The error bar represents the 95% confidence interval. The data points highlighted in red and blue represent the AUC scores of radiologist-measured minimum-JSW and 16-point JSWs, respectively.

Finally, by leveraging the continuous contours of the tibia and femur output by our ResU-Net model, we further divided the joint space into equally spaced regions with several different densities, and hence, the 8, 32, and 64-point JSWs were calculated and subsequently employed for prediction of KL-grade and OA prediction. Figure 5a,b both revealed generally increasing trends of the AUC score as the number of JSWs increases. Specifically, in the classification of KL-grades, the prediction performance levels at 32 points of JSW. This might indicate that 64 points of JSW do not provide more additional information than the 32-point JSWs. On the other hand, the prediction performance increases strictly as a greater number of JSWs are involved. It is noteworthy that in both classifications, the optimal CNN-estimated JSWs yield a similar classification score as the radiologist-measured 16-point JSWs.

## 4. Discussion

In this study, we have proposed a novel deep learning-based approach for automated bone segmentation in the knee joint on radiographic images. Different from the previous works such as BoneFinder [32] and KNEEL [7], which only identify discontinuous landmarks on the bone margin, our proposed deep learning model outputs continuous bone contours, allowing characterization of tibiofemoral joint-space shape in higher resolution [33,34]. Four different prominent neural network architectures, including CUMedVision [16], DeepLabv3 [19], U-Net [21], and ResU-Net-18 [24], designed specifically for image segmentation, were explored and compared for our application. Lastly, the ResU-Net-18 architecture was selected for its high performance (average IoU of 98.9%). We further demonstrated the robust estimation of the JSWs using our trained network, while such estimations do not only agree well with the measurements by radiologists but are also readily applicable for the prediction of KOA severity and progression risk in the future 48-months based on the KL-grading system [10,35].

Instead of merely estimating the minimal JSW in the medial compartment of the tibiofemoral joint, which is known as a common clinical practice in KOA diagnosis, with the continuous contour output by our knee segmentation network, it paves the way for measuring JSWs at multiple fixed locations simultaneously. The experimental results indicated that multiple-JSWs are a significantly better predictor over the single-point minimum-JSW in the classification of KOA severity as well as the prediction of disease progression defined by the KL-grading system. Multiple-JSWs consistently outperform single-point minimum-JSW-based methods on both radiologist measurements and estimated measurements by our model, while radiologist measurements performed better than the estimated measurements by networks slightly but statistically significantly, which might have been caused by less-than-perfect JSW estimations using the network. Moreover, our results also point out that increasing the density of the JSW estimations further enhances the classification performances in both KL-grade and KL-defined radiographic OA progression. It could be explained by the fact that the incorporation of multiple JSW measurements at different locations along the bone contour would provide more information in the characterization of the tibiofemoral joint’s global morphology, which was previously shown to associate with the OA severity [22,36,37]. On top of that, we have further corroborated that joint morphology could also be a valuable predictor of KOA progression.

Previous attempts to apply the traditional computer-vision segmentation approach rely on handcrafted features, such as edge detection filters [1] and active contour methods [6] for segmentation; the former detects every edge on the radiograph using the first-order gradient. However, it could not distinguish the anterior and posterior edge of the tibial articular surface, where the bright bands of subchondral cortical bone of the tibial plateau and femoral condyle instead of the outermost edge visualized on the radiographs are essential for the measurement of JSW [38] (Figure 2). Meanwhile, the latter method’s performance relies heavily on the prior curve parameterization by users to roughly locate the regions of interest, which is usually image-specific, thus leading to a lack of automation during the segmentation process [8,39]. On the other hand, deep neural networks have a large number and automatic feature filter generations, hence allowing the model to learn more complex image details and anatomical structures instead of simple edges and boundaries [33] automatically. Furthermore, this class of models was recently shown to outperform another decision tree-based segmentation technique, BoneFinder [7,40]. Specifically, our deep learning-based bone segmentation approach is superior to the existing approaches in a way that produces a continuous contour of the tibial plateau and femoral condyle rather than discrete landmarks [7,40,41] and is capable of accurately identifying the relevant tibial contour for JSW measurements. This allows preservation of pixel-level boundary information in the tibiofemoral joint, hence, beneficial to the extraction of fine-grained morphological details such as multiple JSWs.

The ResU-Net-18 architecture was selected as the backbone of our deep knee segmentation network owing to its high performance and resistance to overfitting compared to the other three candidates. This network enables the low-level details to be passed across the hidden layers to the final output layer, while its residual blocks extract higher-level features, hence reducing the overfitting problem, as well as ensuring a better fusion of different levels of image features. Additionally, the model adopts atrous convolution, which allows a larger receptive field to be detected [18], thus being beneficial to large image segmentation in our case. On the other hand, the original ResU-Net-50 network was further carefully modified by reducing its number of hidden layers from 50 to 18 to cater to our mono-color, low-variation bone segmentation task; such modification would effectively reduce the risk of over-fitting in the model [24].

As a future plan, external validation of our model on an additional hospital dataset is to be conducted. Moreover, our multiple-JSW approach could also be scaled to the 3-dimensional image modalities, such as computed tomography (CT) images, to characterize the joint space surface landscape for further improvement on the KOA progression prediction.

## 5. Conclusions

In this work, we first employed a deep learning-based approach that automatically detects the bone contours with high accuracy in the knee joint; by leveraging the continuous contours, the JSWs were measured in an automated manner, which was comparable to the radiologist-level measurements. We further demonstrated the capability of our algorithm to provide an acceptable characterization of the global joint-space shape by estimating the JSWs at multiple fixed locations, which is time-consuming, if not impractical, in regular clinical settings. Moreover, for the first time, we found that multiple-JSW measurements are more effective than the commonly used minimum-JSW in classifying the OA severity and the prediction of disease progression. As a result, our method provides a computer-aided tool to the clinical practitioners that could facilitate the KOA diagnosis and prognosis with the fully automated, accurate, and efficient computation of the joint-space parameters.

## Figures and Tables

**Table 1:** Segmentation performance of different deep learning models.

Models	Mean IoU	Validation Loss	Training Loss	Over-Fitting $\left(\frac{Val\;Loss-Train\;Loss}{Val\;Loss}\right)$
CUMedVision	0.973	0.047	0.008	0.830
U-Net	0.594	0.410	0.409	0.002
DeepLabv3	0.989	0.011	0.005	0.545
ResU-Net-18 (ours)	0.989	0.006	0.004	0.333

**Table 2:** KL-grade classification performance using minimum-JSW and 16-point JSWs from the radiologist measurements or CNN-based estimation using XGBoost model. The error represents the 95% confidence interval with a *p*-value of the *t*-test reported for comparison between CNN-based estimation and radiologist measurements.

		Macro Average F1	Average AUC	*p*-Value of AUC Comparison
Minimum-JSW (single-point)	CNN-based Estimation	0.311 (± 0.020)	0.587 (± 0.017)	0.1225
	Radiologist measurement	0.402 (± 0.030)	0.624 (± 0.017)	
16-point JSWs	CNN-based Estimation	0.337 (± 0.027)	0.609 (± 0.022)	0.0063
	Radiologist measurement	0.454 (± 0.024)	0.655 (± 0.014)	

**Table 3:** KL-progression prediction performance using minimum-JSW and 16-point JSWs from radiologist measurements or CNN-based estimation using XGBoost model. The error represents the 95% confidence interval with the *p*-value of the *t*-test reported for comparison between CNN-based estimation and radiologist measurements.

		Average F1	Average AUC	*p*-Value of AUC Comparison
Minimum-JSW (single-point)	CNN-based Estimation	0.484 (± 0.041)	0.554 (± 0.039)	0.898
	Radiologist measurement	0.544 (± 0.032)	0.583 (± 0.040)	
16-point JSWs	CNN-based Estimation	0.480 (± 0.041)	0.551 (± 0.024)	0.1816
	Radiologist measurement	0.562(± 0.044)	0.613 (± 0.018)	

## Data Availability

The dataset presented in this study is available in the Osteoarthritis Initiative (OAI) database (https://data-archive.nimh.nih.gov/oai, accessed on 1 September 2020).
